# Dimethoate Induces DNA Damage and Mitochondrial Dysfunction Triggering Apoptosis in Rat Bone-Marrow and Peripheral Blood Cells

**DOI:** 10.3390/toxics8040080

**Published:** 2020-10-01

**Authors:** Nazia Nazam, Mohammad Iqbal Lone, Abid Hamid, Talal Qadah, Alaa Banjar, Qamre Alam, Mohd Saeed, Waseem Ahmad

**Affiliations:** 1 Gene-Tox Laboratory, Division of Genetics, Department of Zoology, Aligarh Muslim University, Aligarh 202002, UP, India; iqbalzoo84@gmail.com; 2 Cancer Pharmacology Division, CSIR-Indian Institute of Integrative Medicine, Canal Road, Jammu 180001, J&K, India; abidhamid76@gmail.com; 3 Department of Medical Laboratory Technology, Faculty of Applied Medical Sciences, King Abdulaziz University, Jeddah 21589, Saudi Arabia; thqadah@kau.edu.sa (T.Q.); asbanjar@kau.edu.sa (A.B.); 4 Medical Genomics Research Department, King Abdullah International Medical Research Center (KAIMRC), Riyadh 3660, Saudi Arabia; qamar.alam1@gmail.com; 5 Ministry of National Guard—Health Affairs (MNGHA), King Saud bin Abdulaziz University for Health Sciences, Riyadh 22490, Saudi Arabia; 6 Department of Biology, College of Science, University of Ha’il, Hail 2440, Saudi Arabia; mo.saeed@uoh.edu.sa; 7 Center of Excellence in Genomic Medicine Research (CEGMR), King Fahad Medical Research Centre, KAU, Jeddah 21589, Saudi Arabia; waseemahmad2007@hotmail.com

**Keywords:** Dimethoate, comet assay, genotoxicity, cell cycle, mitochondrial membrane potential

## Abstract

Dimethoate (DM) is an organophosphorus (OP) pesticide with wide use in the pest control. Its persistence in crops and soils could possibly cause adverse health consequences in humans as well as other non-target species. Since molecular studies confirming potential genotoxicity of DM have not been previously reported, the acute in vivo toxicological impact was evaluated in Wistar rats. Significant micronuclei induction and metaphase chromosome abnormalities in bone marrow cells exposed to three different DM doses (20, 40 and 60 mg/kg-bw) at multiple treatment durations (24, 48 and 72 h) indicated positive dose response relationship, confirming its genotoxic and cytotoxic potential. Significant mitotic index decrease was seen in dosed animals compared to vehicle control. The study used peripheral blood comet assay, indicating DM-mediated damage to DNA at all exposure levels in a time responsive manner. These assays were found to be an effective, precise, and fast technique with applied value in biomonitoring studies. Cell cycle and apoptosis along with mitochondrial membrane potential (MMP) in flow cytometric analyses confirmed DM exposure decreased MMP, affected the cell cycle, and inflicted DNA damage, which led to cellular apoptosis of leukocytes culminating into immunotoxic effects. The in silico experiments consequently augmented that DM showed acceptable binding energy value for Cyclin A2, suggesting that it could inhibit the cell cycle progression by inhibiting cyclin A2.

## 1. Introduction

The increasing risk associated with hazardous chemical exposure to humans is inevitable. Also, the environmental changes brought about by humans are a potential threat to life. Due to intense usage of pesticides presently, it is crucial to evaluate the genotoxicity induced by chemicals including the organophosphates. This is an alarm and hence, is emphasized by various agencies and investigators [1,2,3]. Dimethoate–DM [0,0- Dimethyl S-(N-methyl carbamoylmethyl) phosphorodithiote], an important organophosphorus (OP) pesticide, is widely used in controlling pests of different cereals, vegetables, fruit, and flies in animal houses in agricultural and sanitary hygiene [4]. Labeled as a “Class II, moderately hazardous” compound, DM has median lethal dose—LD_50_ of 180 to 330 mg/kg-bw in acute toxicity studies in rats [5]. We have established in our previous study on similar DM doses and durations that as with most of the OPs, it also acts as a powerful inhibitor of acetylcholinesterase (AChE) and is neurotoxic [6]. In recent years, the European commission banned the use of Dimethoate for vegetable crops [7]. Even so, it is continuously being used in many countries including India [8,9]. Various epidemiological studies on OP compounds including DM, confirmed their association with non-Hodgkin lymphoma [10] and leukemia [11]. Its genotoxic assessment is of serious concern on account of inconclusive reports on its acute toxicity. Although the positive chromosomal aberration results in mice are questionable [12], the negative cytogenetic reports in rat bone marrow cells [13] were acceptable in the mutagenicity assessment report by US-EPA [14]. However, it is a confirmed genotoxicant, inducing cytogenetic changes including sister chromatid exchanges in human lymphocytes, and micronucleus formation and chromosomal aberration under sub-chronic conditions in mice [15,16]. Importantly, reports on similar doses and durations from our lab established DM-induced oxidant/antioxidant altered status in rats. Heikal and co-workers reported similar results, reinforcing our biochemical toxicity investigations of DM [17]. They also correlated altered biochemical profile and DM-mediated DNA damage in a mammalian model. In the light of investigations by Saquib and co-workers, it is important to note that such oxidative DNA damages or adducts, when not repaired or misrepaired prior to DNA replication, eventually lead to mutations, thereby capable of initiating carcinogenesis [18]. Taking into account the broad use of organophosphates and their associated risk in general and DM particularly, such compounds qualify to be closely examined concerning their toxicity spectrum.

It is enlightening to consider reports on various other organophosphates analyzed for genotoxicity by in vivo studies [19,20]. Chromosomal aberration (CA) with micronuclei (MN) assay is the most consistent genotoxicity evaluation in mammals [21]. Therefore, the MN test (MNT), in combination with the CA assay were performed in our study, together with molecular assays, which can provide indications for clastogenicity. In addition, mitotic index (MI) test was also performed, which is helpful to characterize cell proliferation and to identify compounds with inhibition or induction potential of mitotic progression; therefore being a useful measure of cell proliferation kinetics in animal models [22,23].

Genotoxicity is related to the potential for compound to modify genetic components at sub-toxic concentrations, directing to its diffusion during cell division [24]. A single parameter is insufficient in evaluating the toxicity of a chemical, hence employing multiple genetic tests is a standard protocol. Comet assay is one of test which directly validates DNA damage post-chemical exposure. It is suitable, fast, and practiced globally due to its usefulness in biomonitoring [25].

An effort is made to compare the DNA damage owing to acute toxicity and unravel a mechanistic basis of cellular apoptosis. To the best of our knowledge, toxicity reports on DM necessitate mutagenicity assessment in mammalian test systems, especially when acute toxicity reports are controversial and molecular events leading to such cytogenetic changes are lacking. The current study is thus aimed at elucidating a dose response relationship by assessing (i) the cytotoxicity and genotoxicity of DM in rat bone marrow cells via micronucleus test (MNT), chromosomal aberration (CA) assay, and mitotic index (MI) profile; (ii) DNA strand break by comet assay; (iii) cell cycle alteration to assess apoptotic induction; and (iv) cellular stress due to impaired mitochondrial membrane potential, MMP (ΔΨm) in white blood cells (WBCs) of treated rats, and (v) Molecular docking study of DM with cell cycle regulatory protein.

## 2. Material and Methods

### 2.1. Chemicals

The test compound DM (99% purity), low melting point agarose (LMPA), normal melting agarose (NMA), Na_2_-EDTA, Trizma base, Ethidium bromide (EtBr), propiodium iodide (PI), RNAse, Rhodamine 123 (Rh123), electrophoresis reagents, sodium bicarbonate, phosphate buffered saline (PBS), and tris buffered saline were products of Sigma Aldrich (St. Louis, MO, USA). FACS Lysing Solution was a product of Becton Dickinson (San Jose, CA, USA). Bromophenol blue, Fetal bovine serum (FBS), and Colchicine were obtained from Hi-Media Pvt. Ltd. (Mumbai, India). Cyclophosphamide—CPA (Cyclocel 500) was obtained from Celon Laboratories (Andhra Pradesh, India). May—Gruenwald and Giemsa stain were procured from Merck (India).

### 2.2. Animals—Housing, Acclimatization and Allocation of Groups 

The experimental animals—*Rattus norvegicus* (Wistar strain; 8–10 weeks old; 120 ± 20 gm; males only) were acquired from Indian Institute of Integrative Medicine (IIIM-CSIR) (Jammu, India), quarantined, acclimatized and sacrificed as per standard practice conforming to the University Ethical Regulations. After acclimatization, rats were divided into five groups by stratified randomization with five animals per group. Three groups were exposed to DM while two additional groups served as normal and positive controls. This grouping was maintained for all three sub-lethal doses for acute time periods of 24, 48 and 72 h.

### 2.3. Treatment

As per the method of Musch, the LD_50_ obtained for DM was 200 mg/kg-bw, used in the current study [26]. The three sub-lethal concentrations for DM ranged from low (10% of LD_50_ = 20 mg/kg-bw) to medium (20% of LD_50_ = 40 mg/kg-bw) and high dose (30% of LD_50_ = 60 mg/kg-bw). Cyclophosphamide (0.02%; positive control) and water (vehicle control) were undertaken for comparative study. Distilled water was used to prepare dosing solutions of test agent and injected via intraperitoneal (i.p.) injection [27]. At 24, 48 and 72 h post-dosing, 5 rats per duration were sacrificed and necessary tissues were isolated for different cytogenetic and molecular parameters. For CA assessment, separate experimental lot was used due to colchicine treatment.

### 2.4. Cytogenetic Assays in Bone Marrow Cells

The cytogenetic assays were conducted in compliance with OECD guideline [28].

#### 2.4.1. Micronucleus Test (MNT)

MN assay followed the protocol of Schmid [29]. Immediately after sacrificing the animals, both femurs were removed from the pelvic bone and freed from the extra muscles. After cutting the epiphyses, the bone marrow was collected by gentle flushing with 2 mL FBS. Centrifuge cell suspension at 1000 rpm for 5 min and discarded the supernatant. The pellets obtained were saved in 50 μL fresh FBS. Re-suspended the cell pellet and a small drop was spread onto clean grease-free glass slides and dried in air. By using absolute methanol, the smears were fixed for 10min following the staining by May-Grunwald / buffered Giemsa at pH 6.8. Micronuclei and the ratio of polychromatic erythrocyte (PCE) to normochromatic erythrocyte (NCE) i.e., P/N were scored. The micronucleus frequencies (indicated as mean percent micronucleated cells) were examined by considering the micronucleated PCEs number (MNPCEs) from at least 2000 PCEs/animal. Cytotoxic effect of test compound was evaluated by calculating the PCE:NCE ratio in at least 1000 erythrocytes per animal. For final observation, DPX mounted slides were photographed at 100× with oil immersion (Olympus U—PMTV microscope mounted with optical zoom camera). 

#### 2.4.2. Chromosomal Aberration (CA) Assay

Method of Preston et al. was used for preparing the metaphase chromosomes [30]. An aqueous solution of Colchicine (4 mg/kgbw) was injected intraperitoneally 2 h prior to sacrificing the animal. Pre-warm KCL (0.075 M) solution was used to collect the bone marrow cells followed by incubation at 37 °C for 20 min, and then centrifuge at 1000 rpm for 10 min. After fixing the pellet in cold Cornoy’s fixative dropped onto clean pre-chilled glass slide in 30% ethanol and dried in air. 5% buffered Giemsa (pH 7.0) was used for staining. Chromosomal aberrations were analyzed on properly separated metaphases and finally photographed at 100× (Olympus U—PMTV microscope), using oil immersion. The metaphase cells from approximately 1000 cells per concentration per animal both in exposed and control replicates, expressed in percentage were considered for mitotic indices (MI).

### 2.5. Comet Assay 

Detection of DNA strand break was performed according to protocol of Singh et al. with some modifications [31]. Briefly, blood was collected using Retro-orbital bleeding during euthanasia [32]. 10μL whole blood cells obtained were mixed with 75 µL of 0.5% LMPA and layered onto frosted slides which was pre-coated with NMA. After putting cover slip, slides were kept for 5–10min. After gelling, removed cover slip gently and added a layer of 90 μL of LMPA. After replacing the cover slip, slides were placed on the slide tray (ice packs) till the agarose layer hardened (5–10 min). Following this, removed the cover slip and by gently immersing the slides in lysis buffer embedded cells were lysed. Cells lysis was performed in cold conditions for a minimum of 2 h in lysing solution.

Alkaline conditions (pH > 13) were used to perform the electrophoresis. After lysis, slides were subjected in electrophoretic buffer at 4 °C for 40 min to achieve the DNA denaturation that allow the DNA unwinding and the expression of alkali labile site. Electrophoresis was then carried out at 0.74 V/cm for 40 min (300 mA; 24 volts) at 4 °C by raising or lowering buffer. Thereafter, neutralization buffer (0.4 M Tris, 10 M HCl; pH = 7.5) were employed to wash the slides. Slides were stained by 80 μL of 1× Ethidium Bromide and excess stain was removed by dipping in chilled distilled water. Slides were examined at 40× magnification under fluorescence microscope coupled with a charge coupled device camera (Olympus). 

### 2.6. Flow Cytometry Based Measurement

#### 2.6.1. Isolation of WBCs

WBCs were isolated by following the protocol of Lone et al. [33]. Blood was collected in 0.1% EDTA from retro-orbital plexus of rat, followed by incubation for 30 min. 500 µL of blood was then added to a centrifuge tube with 1 mL FACS lysing solution. The blood was incubated for 10 min and centrifuged (2000 rpm, 10 min) at 25 °C. After discarding the supernatant, pellet was washed in 1mL FACS lysing solution followed by centrifuging at 2000 rpm for 5 min. Repeated this step twice and then pellet was put in 500 µL of PBS, leaving only WBCs. 

#### 2.6.2. Cell Cycle Phase Distribution

Post completion of exposures, PBS was used to wash the harvested cells and fixed in cold ethanol (70%) for 48 h. Cells were digested by RNase (400 μg/mL) at 37 °C for 45 min. After incubating the cells with Propidium iodide (10 μg/mL), examined instantly on flow cytometer FACSAriaII (Becton Dickinson, San Jose, CA, USA). A total of 10,000 events were obtained for cell cycle analysis [34]. DNA content (*x*-axis, PI fluorescence) versus cell counts (*y*-axis) is demonstrated in the FL2 histograms.

#### 2.6.3. Measurement of Mitochondrial Membrane Potential (Ψm) 

Protocol of Majeed and colleagues was employed for flow cytometric measurements of change in Ψm [35]. WBCs were incubated with 5 µM Rh-123 for 60 min at 37 °C. The pellets were re-suspended in 300 µL PBS after washing the cells in PBS at 1500 rpm for 5 min. ΔΨm decrease in 10,000 events was measured by flow cytometer set at 485 nm by analyzing the florescence intensity of Rh-123 in the cells in FITC channel on flow cytometer FACSAriaII (Becton Dickinson, San Jose, CA, USA). Leukocytes were identified via forward and side scatter. 

### 2.7. Molecular Docking Study

The structure of compounds was fetched from PubChem database (CID3082 and CID2907) (http://pubchem.ncbi.nlm.nih.gov) in SDF format (2D-strucuture data) and converted into pdb file, and further were subjected to energy minimization and ligand preparation using Discovery studio suit. 3D structure of cyclin A-2 was acquired from Protein Data Bank (https://www.rcsb.org/) (PDB ID: 4II5). Molecular docking was performed by Autodock 4.2 software using default parameters.

### 2.8. Statistical Analysis

Data were analyzed using analysis of variance (ANOVA) followed by post hoc Tukey multiple comparisons using Statistical Package for Social Sciences (SPSS) Version 20.0. Results were considered significantly different if the P-value was 0.05, unless otherwise stated.

## 3. Results

Compound related mortality did not occur during the study as the doses selected were below the median lethal dose. Animals in experimental groups were comparable to control without any lethality. A reduction in mean body weights of exposed individuals was found in high dose group at all time periods of study; however, these decreases were statistically insignificant. Although the data is not shown, it was generally observed that the mean body weights of control and DM exposed animals were reasonably similar during the study period.

### 3.1. Micronucleus Test (MNT)

**Figure 1:**
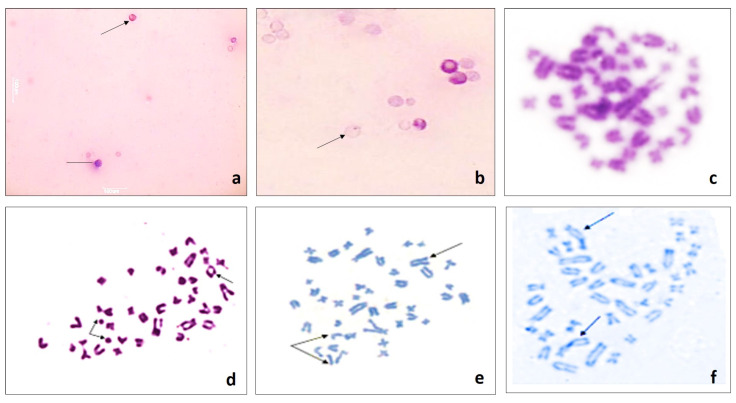
Photomicrograph of *Rattus norvegicus* bone marrow cells treated with different concentration of Dimethoate intraperitoneally. Shown are: (**a**) Polychromatic (arrow) and normochromatic (line) eryhthrocytes; (**b**) MNPCE (arrow) along with PCEs and NCEs and metaphase plates for various chromosomal aberrations (c–f); (**c**) Normal metaphase; (**d**) Ring (arrow) and Break (double arrow); (**e**) Chromosomal gap (arrow) and Acentric fragment (double arrow); (**f**) Dicentric (arrow); (100× oil immersion): Scale bar (**a**,**b**; 10µm) (**c**–**f**; 40 µm).

Representative PCE (blue–purple), NCE (pink) and MNPCE are shown in Figure 1 (a,b). Data obtained for MN assay (Table 1) shows the frequency of MNPCEs among 2000 PCE, indicative of genotoxicity. Increases in the MN frequency in PCEs were noted at all exposure doses and durations of DM. Significant increases (*p* < 0.01; *p* < 0.05) were observed at all DM concentrations, where increases up to 5.6 ± 0.7above the normal control value of 0.27 ± 0.04 were noted. Dose dependent increases in MNPCE frequency were seen while time dependent decreases within a particular group were evident. Highly significant (*p* < 0.001) enhancement in MNPCEs at all exposure durations in the CPA control, reflected the expected outcome of a positive mutagen.

Table 1 also represents the cytotoxic potential of DM in terms of scored PCE among total cells scored (P/N). The P/N ratio showed a mean value close to 0.84 at all durations in the control group. Although the cytotoxicity index decreased slightly (0.740–0.731) in low DM treated rats, and significant (*p* < 0.05) decrease up to 0.711 were noted at two higher concentrations. The decrease in the cytotoxicity indicator reflected a dose and time responsive relationship. The animals exposed to the positive control agent, CPA, resulted in a significant reduction (*p* < 0.01) in the P/N ratio at all time interval.

### 3.2. Chromosomal Aberration (CA)

Structural aberrations induced by varying DM treatment are depicted in Figure 1c–f with emphasis on fragment, deletions, gaps, rings, breaks, and associations. Table 2 enumerates all types of structural aberrations and their frequencies obtained for all animals in control and exposed groups. The observed aberrations were not qualitatively distinct for any treatment; however mean aberration frequency enhanced in a dose-dependent manner in rats treated with DM compared to control. Maximum significant chromosomal aberration of 7.50 ± 1.15 (*p* < 0.01) was observed at the highest concentration 24 h post treatment while damaging effects on chromosomes were less intense at the other two durations of each concentration. However, as the time elapsed, a decrease in the total aberration mean was quite obvious in each treatment group. This reflects anticipated elimination of altered cells caused by DM toxicity. Exposure to a positive mutagen led to highly significant enhancement (*p* < 0.001) in CA frequency, providing experimental proof-of-concept. The study additionally investigated the mitotic index (MI) to describe cell proliferation. A significant dose dependent decrease (*p* < 0.05) in the MI (Table 2), suggests inhibitory effects of DM on cells division.

### 3.3. Comet Assay 

**Figure 2:**
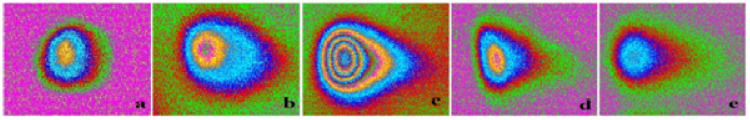
Representative comet images: showing undamaged DNA from control (**a**) with increasing degree of DNA damage as a result of Dimethoate exposure—low (**b**), medium (**c**), high (**d**) and extensively high damage (**e**) exhibiting increasing degree of DNA fragmentation.

**Figure 3:**
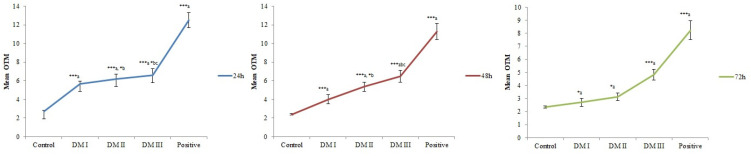
Concentration dependent relationship graphs. Showing the consequence of Dimethoate intoxication on Olive tail moment (OTM) of the comet with increasing doses at multiple time periods assessed by comet assay in rat blood cells. Significant values at * p < 0.05; *** p < 0.001 (One way ANOVA, post hoc Tukey); a (compared to normal control); b (compared to DCV I); c (compared to DCV II); Control (2 mL/kgbw); Positive (40 mg/kgbw CPA); DM I (10 mg/kgbw); DM II (20 mg/kgbw); DM III (30 mg/kgbw).

Representative comet images are illustrated in Figure 2 reflecting an increased degree of damage in peripheral blood cells due to DM treatment (b–e) compared to negative control (a). Various concentration of DM treated rat showed significantly increased DNA migration (*p* < 0.05) compared to normal control (Figure 3), demonstrating an enhancement in DNA damage as a crucial player in median comet OTM for respective drug concentrations and time periods.

Comet parameters scored as the extent of DNA damage measured by (i) Tail Length and (ii) Olive Tail Moment / OTM for each dose and duration group are presented in Table 3. A single dose of 10 mg DM/kgbw for 24 h duration induced extensive damage to DNA, and a nearly 5-fold increase of OTM was seen viz-a-viz negative control (Figure 3). When the dose of DM was doubled, more damage to lymphocyte DNA was observed, showing an OTM rise from 0.81 units in control to 6.19 units. Furthermore, increasing DM concentration reflected highly significant DNA damage registering OTM around 6.59 units. When the damage was measured at 48 h and 72 h post DM treatment, a strict time-dependent decrease was noted (Figure 3). Lymphocytes also showed a highly significant (*p* < 0.001) dose-dependent increase in terms of mean tail length, measured 24 h posttest agent exposure (Table 3). The tail length increased several times from mean value of 22.44 µm at low dose to 30.09 µm for highest DM concentration, with respect to a control value of 2.69 µm. Time dependent pattern of decrease in DNA injury was clear in all dose groups. The images and table clearly indicate that the DNA percentage in comet tail and tail length was significantly enhanced in increasing DM doses. In addition, more DNA moved out of the comet head with increasing the concentration as revealed in Figure 2b–e; on the other hand no tail was found in the control (Figure 2a). Expectedly, high DNA damage in the positive group was seen, as evident by a very significant increase (Table 3) in OTM and tail length in cyclophosphamide treated animals compared to control, hence proving the validation of the study.

### 3.4. Cell Cycle Analysis

**Figure 4:**
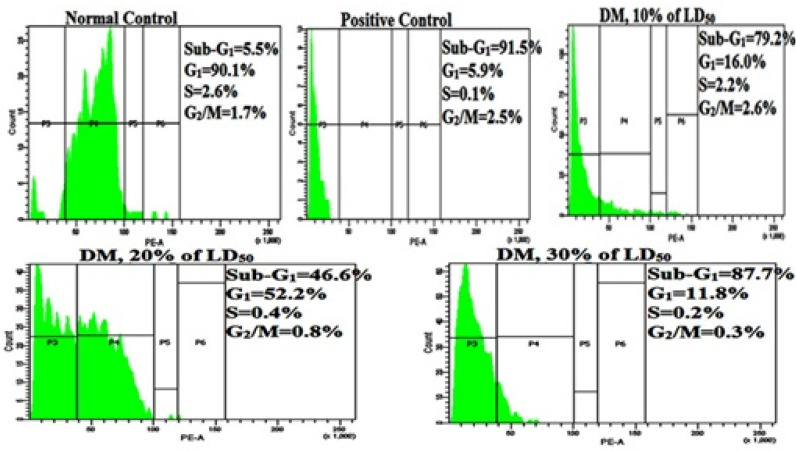
Analysis of cell cycle in lymphocyte cells of Dimethoate treated *R. norvegicus* by flow cytometry. Alteration in progression of cell cycle acquired from one experiment as function of DM treatment. G1, S, G2/M in each image show percentage of cells normal phases of cell cycle, where SubG_1_ exhibit percentage of cells that undergone apoptosis.

The alteration in cell cycle progression obtained from a single animal as function of DM treatment has been shown in Figure 4. G1, S, and G2/M in each micrograph show the cells percentage in normal stages of cell cycle, while SubG_1_ exhibit the cells percentage that have undergone apoptosis. The effect of multiple DM doses and durations on cell cycle phase distribution is summarized in Table 4, confirming maximum apoptotic induction obtained at 24h in a dose response manner. Relative to24h negative control (8.46 ± 0.71), a highly significant increase (*p* < 0.001) in the apoptotic phase from 79.2 ± 2.32 at the lowest, to 83.53 ± 5.37 at the highest DM dose was noted. Within a group, a time dependent decline in the apoptotic cell count was quite conspicuous for all treatments. Hence, DM-induced apoptosis was significant in leukocytes due to dosing, with highest sub-G1 counts at 24 h.

### 3.5. Cyclin A2 Inhibition and Cell Cycle Analysis

**Figure 5:**
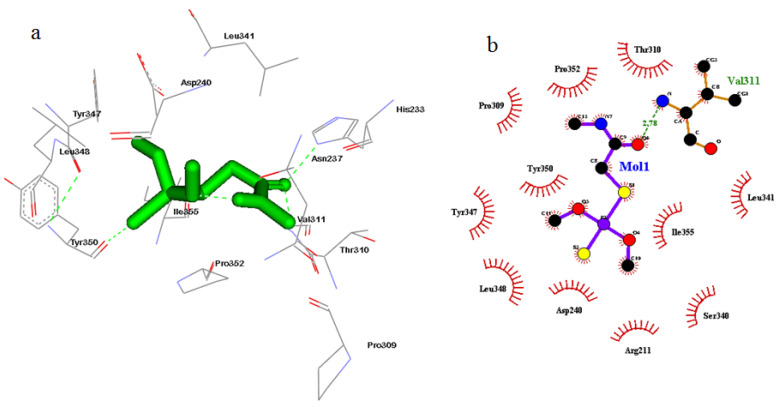
Molecular docking showed strong hydrophobic interaction and hydrogen bonding in “DM-Cyclin A2” (**a**) Interaction of Dimethoate with cyclin A2. The ligand (Dimethoate) has been shown in “stick” representation. (**b**) “Ligplot” of Figure 5a showing bonding interactions.

**Figure 6:**
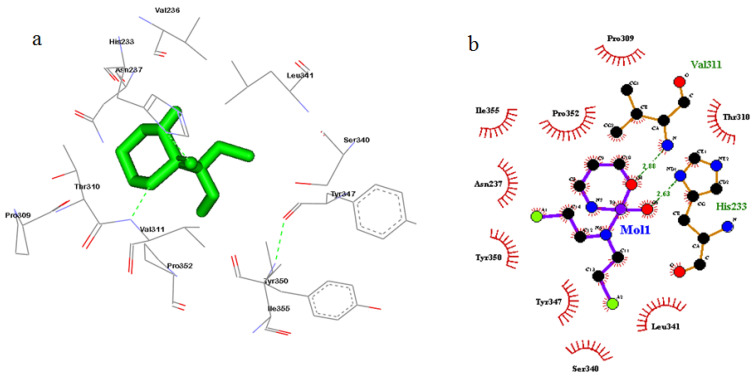
Molecular docking showed strong hydrophobic interaction and hydrogen bonding in “CPA-Cyclin A2” interactions (**a**) Interaction of cyclophosphamide with cyclin A2. The ligand (Cyclophosphamide) has been shown in “stick” representation. (**b**) “Ligplot” of Figure 6a showing bonding interactions.

The catalytic site (CAS) of cyclin A2 was observed to interact with DM through 12 amino acid residues, namely His233, Asn237, Asp240, Pro309, Thr310, Val311, Leu341, Tyr347, Leu348, Tyr350, Pro352 and Ile355 (Figure 5a; Table 5); with CPA through 12 amino acid residues, namely His233, Val236, Asn287, Pro309, Thr310, Val311, Ser340, Leu341, Tyr347, Tyr350, Pro352, and Ile355 (Figure 6a; Table 5). The free energy binding values (ΔG) for “DM-CyclinA2”, and “CPA-CyclinA2” CAS interactions were found to be –5.89 and –5.82 kcal/mol, respectively, and the consequent inhibition constant (K_i_) values were projected to be 259.67 and 54.6 µM, respectively. “Van der Waals”, “Hydrogen bond” and “Desolvation” energy components for DM, and CPA interaction with cyclin A2-CAS were −5.15 and −6.56 kcal/mol, respectively. Additionally, “Electrostatic Energy” components for “DM-Cyclin A2”, and “CPA-Cyclin A2” interactions were found to be −0.16, and −0.13 kcal/mol, respectively. Ligplot analysis reveals that there is a strong hydrophobic interaction and hydrogen bonding in “DM-Cyclin A2”, and “CPA-Cyclin A2” interactions (Figure 5b, Figure 6b).

### 3.6. Analysis of Mitochondrial Membrane Potential

**Figure 7:**
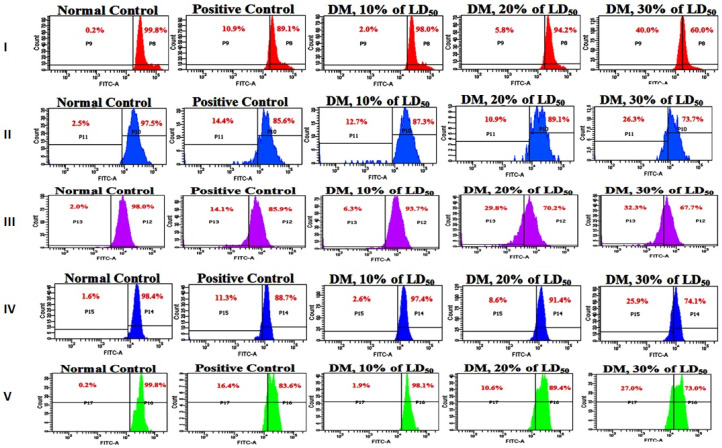
Outcome of Dimethoate treatment on mitochondrial membrane potential (ΔΨm) loss in WBCs of *R. norvegicus*. Characteristic FACS images from an experiment depicting Rh 123 fluorescence as a function of DM concentrations. P8 and P9, P10 and P11, P12 andP13, P14 and P15, P16 and P17 represent percentage of intact and reduced mitochondrial membrane potential in (**I**) Neutrophils, (**II**) Monocytes, (**III**) Lymphocytes, (**IV**) Platelets, (**V**) Eosinophils respectively.

**Figure 8:**
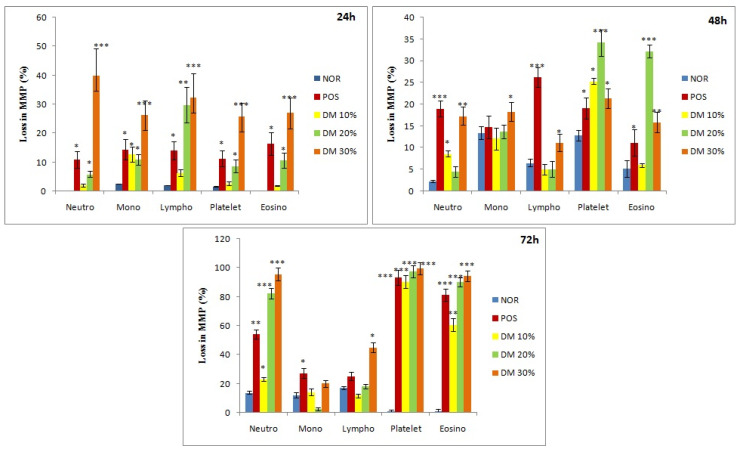
Bar graph representing mean MMP loss in rat WBCs exposed to acute doses of Dimethoate assessed by flow cytometric analysis. * *p* < 0.05, ** *p* < 0.01, *** *p* < 0.001.

A characteristic flow cytometric image obtained from a single experiment depicting Rh 123 fluorescence due to DM intoxication is shown in Figure 7, visibly demarcating MMP in intact and damaged mitochondria in various WBCs. Data generated from such FACS analysis (bar graph in Figure 8) revealed neutrophils and lymphocytes exhibiting maximum MMP loss i.e., greater than 30% at initial exposure time. MMP loss in neutrophils occurred from 2–40% while in lymphocytes from 6.3–32.3% due to increased DM doses, compared to the negative (0.2% and 2%) and positive (10.9% and 14.3%) control ΔΨm values. In other cell types including eosinophils, monocytes and platelets, less than 30% MMP loss (27%, 26.3% and 25.9%) was seen.

At 48 h exposure period (Figure 8), DM showed decrease in MMP loss in all cell types of WBCs still registering around 30% loss in eosinophils. In the 72 h treatment category, however, the effect of DM on MMP loss regained showing greater than 90% membrane potential loss particularly in neutrophils, eosinophils, and platelets as compared to the negative control values of 13.8%, 1.2%, and 1.1%, respectively. Positive control group showed MMP losses of 93.2%, 54% and 81.1% for the respective cell types. Overall, DM confirmed concentration dependent ΔΨm loss in rat WBC, even in 24 h treatment with maximum effect on immune cells, notably neutrophils and lymphocytes.

## 4. Discussion

The conflicting Dimethoate toxicity reports and mutagenicity assays, with serious lack of research on genotoxic effects of Dimethoate in mammalian models, prompted this investigation on *Rattus norvegicus*. Until now, limited acute genotoxicity studies on DM are available; and most of them are inconclusive or unacceptable due to questionable validity of the results or due to a design or reporting deficiency. Only limited numbers of reports are acceptable [13,36]. IARC, therefore, has been unable to classify DM regarding its potential carcinogenicity [3]. In this context, the present investigation holds various implications spanning drug toxicity assessment for human health risk assessment. For cytogenetic responses requiring DNA replication—24, 48 and 72 h sampling times were used throughout our study in agreement with previous work by Bakheet and co-workers [37]. Also, a minimum of three dose levels along with negative and positive control groups were used to detect early DNA damage, as per Hartmann and colleagues [38]. The durations chosen are further justified considering earlier reports as they allow an appropriate window period to detect clastogen and spindle poisons [39,40]. Thus, the rationale of the study and the significant genetic alterations measured validates the effectiveness of the study and would aid in defining DM exposure concentrations for the chemical safety assessment together with other toxicological endpoints.

The rodent erythrocyte micronucleus and chromosomal aberration assay are markers of early biological effects of carcinogens. Hence, International regulatory agencies recognize MN and CA tests as most successful and reliable assays for genotoxic carcinogens [41,42]. The MN assay in our study reflected bone marrow suppression (expressed in terms of P/N), which was a dose and time dependent response. Mechanisms such as direct cellular toxicity, MN induction, and profound DNA damage leading to apoptosis or cell death may be contributing factors to our results on bone marrow inhibition. The increasing MNPCE frequency followed strict dose dependence while the increase in MNPCEs frequency at 24 h post-treatment with a gradual decrease at other two durations is noteworthy. This time response reflects bone marrow turnover i.e., removal of damaged cells from the bone marrow and replacement by newly formed undamaged cells. 

Nevertheless, May-Gruenwald/Giemsa staining in MN assay could not distinguish between micronuclei-containing fragments or whole chromosomes, hence chromosome aberration assay was performed further. The rate of bone-marrow proliferation (expressed in terms of MI) was significantly inhibited in DM-treated groups relative to control. The decrease in MI (being critical in determination of cell division rate) indicates a slower cells progression from **“**S**”** to **“**M**”** phase of cell cycle further corroborated by our cell cycle experiments. These findings confirmed that the test doses induced cytotoxic effect, with recovery and repairing ability in mitosis at later exposure times. Also, DM demonstrated direct interaction with DNA leading to damage of genetic material evident by various structural chromosomal aberrations such as break, deletions, gap, rings and acentric fragments. These aberrations caused by improperly repaired DNA breaks confirms the clastogenic potential of Dimethoate. It is also crucial to note that when cells with few chromosomal abnormalities persists for longer, their further division could be associated with mutations that could be passed on to the next generation through clonal expansion, hence capable of manifesting the damage in the genome, thus the study holds promising results from heritable genetics view point too. Information regarding DNA damages obtained by cytogenetic methods were quite promising. Hence, DNA damage at the individual cell level was measured using comet assay—a potentially useful in vivo genotoxicity test on account of its rapidity, simplicity to perform, higher sensitivity and specificity [43,44]. Our comet data on rat peripheral blood explain significant dramatic increases in DNA damage through enhancement in the comet tail (DNA fragments migration) from the comet head (nucleus). This reflects DNA damage in DM exposed cells strongly correlating with a feature of cellular apoptosis or necrosis [45]. Jagetia and colleagues also suggests that the DNA damage buildup is the characteristic of cell death [46]. The enhanced migration of DNA in peripheral blood cells found after DM exposure could well be accounted to cytotoxicity and genotoxicity induced by DM. The enhanced olive tail moment in the current investigation could possibly be due to direct induction of DNA strand breaks or DNA modification ending into strand-breaks. Schipler and Iliakis also support the fact that chemically induced DNA strand breaks or their byproducts could probably be due to DNA bases modifications or sugar phosphate backbone [47]. The increased OTM is also expected from topoisomerase II stabilization; major factor which establishes break in DNA strand on its interaction with free radicals [48], which may have been enhanced by DM. Free radical mediated oxidative stress has already been established in similar experiments done with DM reported from our lab [49]. The stimulation of DNA damage by DM cannot be attributed to a single mechanism but is likely the interplay of several different mechanisms. Loss of damaged cells, DNA repair of living cells and xenobiotic detoxification/biotransformation by metabolizing enzyme cytochrome P450 could contribute to the DNA repair and influence the comet assay results, which are also expected to play role in our investigation [50].

Apoptosis, a distinctive pathological process, may lead to disease if it is mis-regulated [51]. Therefore, apoptosis with associated signaling pathways that control or restrain apoptosis, is important for clinical and biomonitoring rationales. Hence analysis of PI-stained apoptotic nuclei—a fast, consistent and reproducible estimate of apoptosis was employed in our study using flow cytometry. To the best of our knowledge, understanding with respect to cytotoxic effects of DM on WBC functions and the associated mechanisms of action was lacking; therefore, to elucidate potential mechanistic action of DM, further investigation was undertaken. Analysis of cell cycle established decreased G_2_/M cell cycle arrest at either duration or doses of DM, suggesting the DNA repair process was insufficient to repair the DNA damage incurred. Consequently, transition of cells from G_2_/M phase to the sub-G1 or apoptotic phase was evident in a dose response manner. The appearance of a Sub-G_1_ peak, characteristic of apoptosis, supports this shift. Our next aim was to unravel the probable target of Dimethoate regulating apoptosis in the present case. Therefore, further docking experiment were performed, in which free energy of binding determine the strength of interaction between a ligand and target protein [52,53]. The lowest binding energy is the output of the efficient binding of drug/ligand to its active site of the target protein [54,55]. Accordingly, the docked ligands (DM and CPA) exhibited acceptable binding energy values for Cyclin A2. These data suggest that DM and CPA could inhibit the cell cycle progression by inhibiting cyclin A2. Somatic cells are found express Cyclin A2 that regulates both the G1/S and the G2/M checkpoint [56]. Therefore, it was a cue in our case that cyclin A2 could be involved in mediating apoptosis upon Dimethoate treatment. Further when molecular docking was performed, strong hydrophobic interaction and hydrogen bonding between catalytic site of cyclin A2 with Dimethoate was observed. Thus, with in silico results we predict Cyclin A2 inhibition due to interaction of Dimethoate with this particular cyclin. Hence, to arrest DNA damaged cells in the M phase, cyclin A2 might be insufficient subsequently leading to cellular death and hence an upsurge in Sub-G1 apoptotic population was observed. 

Although flow cytometry-based assays are associated with limitations regarding detection of lysed or damaged cells [57], the major advantage is their high sensitivity at the single cell level. A positive correlation between cell cycle deregulation and various neoplastic diseases is well established [58]. Therefore, our assessment of cell cycle which confirms DM-induced apoptosis in leukocytes is a pointer for further investigation. From the physiological viewpoint, apoptotic cells have been seen to contribute to immune suppression significantly [59]. Therefore, the action of DM in apoptotic events is speculated to decrease the functional leukocytes number simultaneously decreasing other blood cell counts leading to lower immune function levels strengthened by similar effects with other chemicals by Munson and team [60]. Future research targeting other molecular mechanisms due to DM effects on these immune cells is thus needed.

The MMP decrease due to DM treatment reflects DM as an uncoupler of oxidative phosphorylation leading to decreased mitochondrial membrane potential in a concentration dependent manner. It could thus be believed that at initial duration, i.e., 24 h all DM doses is more toxic to mitochondrial oxidative phosphorylation. Loss of MMP is indicative of mitochondrial membrane leakage and permeability, and release of cell death mediators triggering the final apoptotic damage. This is one of the many crucial pathways for apoptosis of the cells of immune system as confirmed by various researchers [61,62]. Thus, the remarkably decreases Ψm in the cells of the immune system in a dose dependent manner at all durations in this study supports the hypothesis that mitochondrial driven apoptosis are caused by Ψm decrease in the cells [63].

We also interpret the overall findings of this study focused on DM-mediated DNA damage through the biochemical approach. The incurred DNA damage could be due to the interaction of DM or its metabolite with cellular DNA, due to reactive oxygen species (ROS) production leading to strand breaks. We already established role of ROS in our earlier research on DM acute effects leading to oxidative stress [49]. A comparable study on another organophosphate corroborates our findings [63]. Yet another conclusion of this study is that damage due to oxidative stress accumulates more in mitochondria due to continuous leak of electrons from the respiratory chain leading to ROS generation. DNA, lipids and mitochondrial proteins are modified by oxidative damage leading to failure of mitochondrial bioenergetics and finally apoptotic cell death [64] as evident from our comprehensive study on Dimethoate-induced toxic effects. Carlson and Ehrich also support that the sub-cellular events that initiate cytotoxicity (confirmed by cytogenetics indices presently)—resulting in the apoptosis—are associated with functional alterations in mitochondrial membranes exposed to organophosphate compounds [65]. Changes in mitochondrial potential measured with cationic fluorescent probe Rh123 in the present study points to the role of oxidative stress in toxicity of DM. Dimethoate could inhibit the cell cycle progression by inhibiting cyclin A2 The effect of DM on the activation of genes related to apoptosis (p53, caspase 3 and 9) is expected and potential mechanism of the toxic action of ROS is highlighted. Also, the positive control mutagen used in the study produced the expected cytogenetic and molecular responses, and the results agreed with earlier studies [66,67]. 

The most important application of the findings of this study is for safeguarding the human population. Most pesticides have confirmed carcinogenic effects in humans via a variety of mechanisms including genotoxicity, chromosomal aberrations, immunotoxicity, oxidative stress etc. [68]. Organophosphorus pesticides specifically have been seen to play a role in the pathology and chromosomal damage, together with epidemiologic associations with cancer in humans [69,70]. Thus, the study is helpful in cytogenetic biomonitoring population study. The use of biomarkers in the present study represents intermediate steps in the pathway from exposure to disease, therefore can be applied in cancer risk estimation in human populations. The smaller size and lower costs of studies together with the potential for early detection of risk in exposed populations are some practical advantages. We also recommend use of biomarkers such as MN, CA and comet assay for such purposes [71]. Furthermore, the molecular markers (used presently) also confirms that cells have been exposed to mutagens/carcinogens. Thus, the finding may help in guarding against genetic hazards to the human population and the environment through judicious and careful use of this particular organophosphate in agricultural and non-agricultural arenas.

## 5. Conclusions

Cytogenetic endpoints indicate that DM administration in mammalian model has cytotoxic and genotoxic effect confirmed by micronuclei formation, production of structural chromosome aberrations, bone marrow suppression and increased DNA damage indices in the comet assay. Molecular analysis confirmed the potential of Dimethoate to induce cytotoxicity as well as DNA toxicity, highlighted by appearance of the SubG1 apoptotic peak in the cell cycle and ΔΨm dysfunction in treated rats. In silico analysis augments cell cycle alteration by this mutagen could possibly be by the inhibition of cyclin A2. All together these data substantiate the sensitivity of the experimental protocol followed in the detection of DNA damaging effects. We conclude that DM exposure lead to cytogenetic damage, and its genotoxic and immunotoxic potential is confirmed from the present endeavor. 

## Figures and Tables

**Table 1:** Mean percent count of micronucleated Polychromatic and Normochromatic erythrocytes (MNPCE and MNNCE) for bone marrow cells of *R. norvegicus* treated with dimethoate along with respective controls at different time periods.

Group and Dose	Exposure (h)	MNPCE (Mean% ± SD)	P/N
**Normal Control** **(Distilled water: 2 ml/kgbw)**	24	0.27 ± 0.04	0.840
	48	0.29 ± 0.07	0.844
	72	0.21 ± 0.01	0.847
**Positive control** **(Cyclophosphamide: 40 mg/kgbw)**	24	10.6 ± 0.81 ***	0.604 *
	48	8.2 ± 0.66 ***	0.529 **
	72	7.1 ± 0.43 ***	0.581 **
**Low Dose** **(DM: 20 mg/kgbw)**	24	4.4 ± 0.51 **	0.740 *
	48	4.0 ± 0.44 *	0.736 *
	72	3.6 ± 0.50 *	0.731 *
**Medium Dose** **(DM: 40 mg/kgbw)**	24	5.2 ± 0.58 **	0.728 *
	48	4.8 ± 0.86 **	0.715 *
	72	4.1 ± 0.73 *	0.711 *
**High Dose** **(DM: 60 mg/kgbw)**	24	5.6 ± 0.70 **	0.725 *
	48	4.9 ± 0.74 **	0.721 *
	72	3.8 ± 0.80 *	0.718 *

Statistically significant values at * *p* < 0.05, ** *p* < 0.01, *** *p* < 0.001 (One-way ANOVA, post hoc Tukey).

**Table 2:** Number and mean percentages of chromosomal aberrations and mitotic indices mean percentages in *R. norvegicus* bone marrow cells after multiple hours of Dimethoate exposure.

Duration (h)	Group	Structural Aberrations							Total Aberrations	MI
		Brk	Del	Frag	Gp	Dcn	Ring	Assoc	(Mean ± SD)	(Mean ± SD)
24	Normal Control	1	0	2	0	0	2	0	0.83 ± 0.40	2.65 ± 0.09
	Positive Control	14	23	26	20	25	8	9	17.50 ± 3.33 ***^,a^	1.52 ± 0.05 **^,a^
	D I	2	4	6	8	8	2	4	5.66 ± 1.03 **^,a^	2.19 ± 0.21 *^,a^
	D II	4	4	3	8	12	6	6	5.83 ± 1.69 **^,a^	2.16 ± 0.16 *^,a^
	D III	6	6	8	7	10	4	4	7.50 ± 1.15 **^,a,^*^,b^	1.92 ± 0.61 *^,a^
48	Normal Control	0	1	1	0	0	0	0	0.33 ± 0.21	2.95 ± 1.06
	Positive Control	12	18	21	16	18	6	7	13.66 ± 2.56 ***^,a^	1.56 ± 0.05 **^,a^
	D I	9	5	12	6	3	6	5	5.33 ± 1.43 **^,a^	2.04 ± 0.15 *^,a^
	D II	3	4	16	7	8	5	6	5.16 ± 1.74 **^,a^	1.98 ± 0.08 *^,a^
	D III	7	5	14	8	7	9	7	6.83 ± 1.90 **^,a^	1.97 ± 0.12 *^,a^
72	Normal Control	0	0	1	0	0	0	0	0.16 ± 0.14	2.71 ± 0.08
	Positive Control	9	15	19	13	14	3	4	10.66 ± 2.61 ***^,a^	1.63 ± 0.03 **^,a^
	D I	2	4	4	5	2	1	0	2.17 ± 0.83 *^,a^	2.07 ± 0.10 *^,a^
	D II	5	2	5	7	2	1	1	2.67 ± 0.75 *^,a^	2.05 ± 0.14 *^,a^
	D III	8	3	3	6	2	2	1	3.16 ± 0.71 *^,a^	1.95 ± 0.04 *^,a^

Statistically significant values at *p < 0.05, ** *p* < 0.01, *** *p* < 0.001 (One-way ANOVA, post hoc Tukey); ^a^ (compared to normal control), ^b^ (compared to DM I). Normal Control (Distilled water, 2 mL/kgbw), Positive control (40 mg/kgbw), DM I (20 mg/kgbw), DM II (40 mg/kgbw), DM III (60 mg/kgbw); Brk—Break, Del—Deletion, Frag—Fragment, Gp—Gap, Dcn—Dicentric, Rng—Ring, Assoc—Association.

**Table 3:** Effect of multiple sub-lethal doses of Dimethoate acute exposure on comet assay parameters analyzed in whole blood of *Rattus norvegicus.*

Groups	Time (h)	Olive Tail Moment	Tail Length (µm)
**Control**	24	0.81 ± 0.16	2.69 ± 0.58
	48	0.65 ± 0.09	2.39 ± 0.52
	72	0.56 ± 0.08	2.35 ± 0.49
**Positive**	24	12.52 ± 0.81 ***^,a^	51.01 ± 3.71 ***^,a^
	48	11.28 ± 0.84 ***^,a^	40.91 ± 2.86 ***^,a^
	72	8.23 ± 0.73 ***^,a^	38.51 ± 1.05 ***^,a^
**DM I**	24	5.65 ± 0.33 ***^,a^	22.44 ± 2.07 ***^,a^
	48	4.01 ± 0.48 ***^,a^	19.26 ± 1.00 *^,a^
	72	2.71 ± 0.31 *^,a^	16.16 ± 1.35 *^,a^
**DM II**	24	6.19 ± 0.52 ***^,a,^*^,b^	24.37 ± 1.66 ***^,a,b^
	48	5.35 ± 0.48 ***^,a,^*^,b^	25.07 ± 1.87 ***^,a,b^
	72	3.13 ± 0.28 *^,a^	21.12 ± 1.45 ***^,a^
**DM III**	24	6.59 ± 0.67 ***^,a,b,c^	30.09 ± 2.86 ***^,a,b^
	48	6.46 ± 0.64 ***^,a,b,c^	27.63 ± 3.12 ***^,a,c,^**^,b^
	72	4.84 ± 0.41 **^,a^	20.37 ± 1.59 *^,a,c^

Values are Mean ± SD, * *p* < 0.05; ** *p* < 0.01; *** *p* < 0.001 (One way ANOVA, post hoc Tukey); ^a^ (compared to normal control); ^b^ (compared to DM I); ^c^ (compared to DM II). Control (2 mL/kgbw water); Positive (40 mg/kgbw CPA); DM I (20 mg/kgbw); DM II (40 mg/kgbw); DM III (60 mg/kgbw).

**Table 4:** Flow cytometry-based cell cycle phase distribution analysis with propidium iodide staining in rat WBCs treated with different doses of Dimethoate at various durations.

Groups and Dose	Duration	Sub-G1	G1	S	G2/M
**Control**	24 h	8.46 ± 0.71	79.93 ± 2.23	7.33 ± 0.89	4.16 ± 0.46
	48 h	9.36 ± 0.82	85.81 ± 3.21	3.2 ± 0.03	2.1 ± 0.36
	72 h	5.53 ± 0.72	90.14 ± 3.89	2.86 ± 0.02	1.6 ± 0.04
**Positive**	24 h	91.3 ± 4.12 ***^,a^	6.06 ± 1.26	0.2 ± 0.01	2.53 ± 0.06
	48 h	76.3 ± 4.16 ***^,a^	21.33 ± 2.41	0.06 ± 0.01	1.3 ± 0.2
	72 h	98.4 ± 6.01 ***^,a^	0.87 ± 0.03	0.33 ± 0.11	0.4 ± 0.02
**DM I**	24 h	79.2 ± 2.32 ***^,a^	15.66 ± 1.28 **^,a^	2.2 ± 0.03	2.8 ± 0.23
	48 h	21.13 ± 1.73 *^,a^	77.4 ± 3.42	0.53 ± 0.22	1.1 ± 0.21
	72 h	18.9 ± 2.62 *^,a^	81.06 ± 4.29	0.33 ± 0.02	0.43 ± 0.16
**DM II**	24 h	75.5 ± 3.27 ***^,a^	19.1 ± 1.21 **^,a^	1.6 ± 0.11	2.8 ± 0.05
	48 h	37.13 ± 2.12 **^,a^	57.6 ± 2.02 *^,a^	2.4 ± 0.54	2.8 ± 0.26
	72 h	26.3 ± 2.63 *^,a,b^	72.16 ± 4.06	0.47 ± 0.14	0.8 ± 0.03
**DM III**	24 h	83.53 ± 5.37 ***^,a^	15.2 ± 0.38 ***^,a^	0.33 ± 0.11 **^,a^	0.83 ± 0.13 **^,a^
	48 h	39.2 ± 2.38 *^,a^	54.83 ± 2.58 *^,a^	6.1 ± 0.38 *^,a^	0.4 ± 0.11 **^,a^
	72 h	37.8 ± 3.01 *^,a,b,c^	55.63 ± 1.04 *^,a^	5.2 ± 0.25 *^,a^	2.3 ± 0.05

Values are Mean ± SD, * *p* < 0.05; ** *p* < 0.01; *** *p* < 0.001 (One way ANOVA, post hoc Tukey); ^a^ (compared to normal control); ^b^ (compared to DM I); ^c^ (compared to DM II). Control (2 mL/kgbw water); Positive (40 mg/kgbw CPA); DM I (20 mg/kgbw); DM II (40 mg/kgbw); DM III (60 mg/kgbw).

**Table 5:** Amino acid residues of Cyclin A2 involved in respective ligand interactions.

Compound	Target	Binding Energy (kcal/mol)	Interacting Amino Acid Residues
Dimethoate		−5.89	His233, Asn237, Asp240, Pro309, Thr310, Val311, Leu341, Tyr347, Leu348, Tyr350, Pro352, Ile355
	Cyclin A2		
Cyclophosphamide		−5.82	His233, Val236, Asn287, Pro309, Thr310, Val311, Ser340, Leu341, Tyr347, Tyr350, Pro352, Ile355
